# Reimbursed Medication Adherence Enhancing Interventions in European Countries: Results of the EUREcA Study

**DOI:** 10.3389/fphar.2022.892240

**Published:** 2022-06-17

**Authors:** Tamás Ágh, Maja Ortner Hadžiabdić, Kristina Garuoliene, Anne Gerd Granas, Emma Aarnio, Enrica Menditto, João Gregório, Pilar Barnestein-Fonseca, Vildan Mevsim, Przemysław Kardas, European Network to Advance Best Practices and Technology on Medication Adherence (ENABLE)

**Affiliations:** ^1^ Syreon Research Institute, Budapest, Hungary; ^2^ Center for Health Technology Assessment and Pharmacoeconomic Research, University of Pécs, Pécs, Hungary; ^3^ Centre for Applied Pharmacy, Faculty of Pharmacy and Biochemistry, University of Zagreb, Zagreb, Croatia; ^4^ Pharmacy Center, Institute of Biomedical Science, Faculty of Medicine, Vilnius University, Vilnius, Lithuania; ^5^ Section for Pharmaceutics and Social Pharmacy, Department of Pharmacy, University of Oslo, Oslo, Norway; ^6^ Norwegian Centre for E-health Research, University Hospital of North Norway, Tromsø, Norway; ^7^ School of Pharmacy, University of Eastern Finland, Kuopio, Finland; ^8^ CIRFF, Center of Pharmacoeconomics and Drug Utilization Research, Department of Pharmacy University of Naples Federico II, Naples, Italy; ^9^ CBIOS, Universidade Lusófona’s Research Center for Biosciences & Health Technologies, Lisboa, Portugal; ^10^ CUDECA Institute for Training and Research in Palliative Care, CUDECA Hospice Foundation, Málaga, Spain; ^11^ Department of Family Medicine, Faculty of Medicine, Dokuz Eylul University, Izmir, Turkey; ^12^ Medication Adherence Research Centre, Department of Family Medicine, Medical University of Lodz, Lodz, Poland

**Keywords:** medication adherence, persistence, intervention, reimbursement, health economics, health policy

## Abstract

**Introduction:** Current literature lacks detailed understanding of the reimbursement framework of medication adherence enhancing interventions (MAEIs). As part of the ENABLE COST Action, the EUREcA (“EUropen REimbursement strategies for interventions targeting medication Adherence”) study aimed to provide an in-depth overview of reimbursed MAEIs currently available in European countries at national and regional levels and to pave the way for further MAEIs to be implemented in the future.

**Methods:** A web-based, cross-sectional survey was performed across 38 European countries and Israel. The survey questionnaire was developed as a result of an iterative process of discussion informed by a desk review. The survey was performed among invited ENABLE collaborators from June to July 2021. Besides descriptive analysis, association between country income and health care expenditure, and the availability of reimbursed MAEIs were also assessed.

**Results:** The survey identified 13 reimbursed MAEIs in nine countries: multi-dose drug dispensing (*n* = 5), medication review (*n* = 4), smart device (*n* = 2), mobile application (*n* = 1), and patient education (*n* = 1). The median GDP per capita of countries having ≥1 reimbursed MAEI was significantly higher compared to countries having no reimbursed adherence intervention (33,888 EUR vs 16,620 EUR, respectively; *p* = 0.05).

**Conclusions:** Our findings highlight that to date only a small number of MAEIs have been reimbursed in European countries. Comprehensive health technology assessment recommendations and multi-stakeholder collaboration could help removing barriers related to the implementation and reimbursement of MAEIs.

## Introduction

According to the estimation of the World Health Organization (WHO), adherence to long-term pharmacotherapies averages only 50% (WHO, 2003). Medication non-adherence has a serious negative impact on health outcomes and results in increased health care utilization and costs (Breekveldt-Postma et al., 2008; Cutler et al., 2018; Kleinsinger, 2018; Mongkhon et al., 2018; Inotai et al., 2021). It should be also noted that the trend of accelerated aging society in the 21^st^ century increases the burden of multimorbidity and polypharmacy and consequently the likelihood and negative consequences of poor adherence (Midao et al., 2018; Kardas et al., 2021; Kurczewska-Michalak et al., 2021).

Several medication adherence enhancing interventions (MAEIs) - including many innovative technologies (e.g., smart devices, mobile applications) - have been developed in the last decade which may greatly improve suboptimal adherence to therapies and hence, therapeutic outcomes (Salema et al., 2011; Nieuwlaat et al., 2014; Costa et al., 2015; van Driel et al., 2016; Blakey et al., 2018; Godinho et al., 2020; Zijp et al., 2020; Gohil et al., 2021; Whiteley et al., 2021). The need for these technologies became increasingly important during the COVID-19 pandemic (Agh et al., 2021). However, currently MAEIs are mainly used within clinical research settings and little is known about their implementation in routine clinical practice (Zullig et al., 2019; Kostalova et al., 2022).

To our knowledge, there is a gap in the scientific literature with regards to the implementation, health technology assessment (HTA), policy regulation and reimbursement of MAEIs. In 2018, the Organisation for Economic Co-operation and Development (OECD) identified four enablers for improving medication adherence at the system level, such as (i) acknowledge (“to acknowledge that medication non-adherence harms health and increases healthcare costs”), (ii) inform (“to systematically monitor adherence”), (iii) incentivise (“to make changes in financial incentives for providers and patients”), and (iv) steer and support (“adherence begins with a patient and a prescribing clinician and a dispensing pharmacist who should all be supported by other health system stakeholders”) (Khan and Socha-Dietrich, 2018). Nevertheless, neither this OECD study (Khan and Socha-Dietrich, 2018) nor other key publications on this topic (Nieuwlaat et al., 2014; WHO, 2014) did provide any recommendation on the implementation and reimbursement of MAEIs. Beside the above listed factors, barriers to implementation may also include the limited evidence on the cost-effectiveness of these interventions (Elliott et al., 2005; Simon-Tuval et al., 2016). Moreover, successful implementation of these innovative technologies in daily practice is further hampered by significant differences between healthcare systems, reimbursement pathways and policy regulations across countries which makes the issue of transferability of MAEIs highly relevant (Khan and Socha-Dietrich, 2018).

To overcome challenges related to implementing MAEIs, on October 2020 the European Network to Advance Best practices and technoLogy on medication adherencE (ENABLE, COST Action 19132) was launched. ENABLE is a 4-years research initiative funded by the European Commission that is expected to catalyze research, policy, and implementation regarding MAEIs across healthcare systems in all European countries and Israel (van Boven et al., 2021). As part of the ENABLE research project, the objectives of this study were to provide an in-depth overview and critical assessment of reimbursed MAEIs in European countries at national and regional levels in order to identify good practice models and to pave the way for further MAEIs to be implemented in the future.

## Material and Methods

### Study Design

An anonymous, web-based, cross-sectional survey, called the “EUropean REimbursement strategies for interventions targeting medication Adherence” (EUREcA), was performed across 38 European countries (i.e., Albania, Austria, Belgium, Bosnia and Herzegovina, Bulgaria, Croatia, Cyprus, Czech Republic, Denmark, Estonia, Finland, France, Germany, Greece, Hungary, Iceland, Ireland, Italy, Latvia, Lithuania, Luxemburg, Malta, Moldova, Montenegro, the Netherlands, North Macedonia, Norway, Poland, Portugal, Romania, Serbia, Slovakia, Slovenia, Spain, Sweden, Switzerland, Turkey, and the United Kingdom) and Israel. The target population of the survey was limited to members of ENABLE including academics with medical or pharmaceutical backgrounds, healthcare providers and health economists. Ethical issues for this study were governed by the Ethical Committee of the Medical University of Lodz, Poland. According to the policy of that Commission, non-experimental studies are not a subject to ethical approval procedure, and hence, such an approval was not needed. Each participant was requested to provide a written, online recorded informed consent before completing the survey. No personal data was stored in relation to this survey. The study was reported according to the Checklist for Reporting Results of Internet E-Surveys (CHERRIES) (Eysenbach, 2004).

### Questionnaire Development

The primary outcome of the survey was a better understanding on the available reimbursed MAEIs across European countries. In relation to the aim of this study, MAEI was defined as “any structured intervention aiming to help patients to make optimal use of their pharmacotherapy”. Interventions of interest could be reimbursed/financed by public funds, pharma companies, patient organizations or any other organizations implemented at national and regional levels targeting any kind of pharmacotherapy (regardless of health condition). The survey questionnaire was developed as a result of an iterative process of discussion and consensus among the authors informed by a desk review. The draft questionnaire was validated by four external adherence experts with respect to the face validity and the technical functionality of the online questionnaire. Finally, the questionnaire contained one question on informed consent, three questions on demographic data, nine questions per intervention, allowing for maximum three reimbursed MAEIs per respondent per country, one question on data regarding reimbursed MAEIs planned to be introduced in the next 24 months and one question on any other relevant information. The majority of questions were closed, multiple-choice questions or “yes”/“no” questions; there were only two open-ended questions. A copy of the survey questionnaire can be seen in Supplementary Figure S1.

### Data Collection

The EUREcA survey was posted on SurveyMonkey.com (www.survey-monkey.com) on 15th of June 2021. The survey was not open for the general public. A unique link to access the web-based survey was sent by email to ENABLE members (*n* = 85). At the beginning of the survey, before giving informed consent, all participants were informed about the objectives of the survey, the use and storage of the data and the length of time of the survey. The online questionnaire was distributed over 23 pages. The average time required to complete the survey was estimated to be 20 min. The survey was open until 20th of July 2021; reminders were sent weekly to all invited ENABLE collaborators. No incentives were offered to participants for completing the survey. Online surveying system settings were set to prevent multiple entries from the same individual IP address.

### Data Analysis

As the first step of data synthesis, a completeness check was conducted to ensure that adequate responses were received. Only data on interventions with complete set of information (i.e., answers were provided to all questions) were included in the analysis. In case of more than one respondent from a country, survey results were sent to the ENABLE country representatives for clarifications and data validation.

Data on the identified reimbursed MAEIs were presented in a descriptive way. Mann-Whitney *U* test was used to assess the differences in country income (i.e., real gross domestic product [GDP] per capita in 2019 EUR) (Eurostat, 2022) and health care expenditure data (i.e., health care expenditure per capita in 2019 EUR) (OECD, 2020) between countries reporting ≥1 vs no reimbursed MAEI. In all statistical analyses, the significance level was set at 0.05. Statistical analyses were performed using R software (The R Foundation for Statistical Computing, Vienna, Austria; version 4.1.2).

## Results

### Survey Participants

Fifty-four participants (survey response rate = 64%) covering all 39 ENABLE countries (1, 2, and three respondents from 26, 11, and two countries, respectively) completed the survey (Table 1). Sixty-seven percent (*n* = 36) of respondents had academic background (i.e., medical or pharmaceutical sciences) and 76% (*n* = 41) of participants had more than 10 years of work experience.

**TABLE 1:** General characteristics of survey participants.

Country	Number of Survey Participants	Primary Field of Work (Work Experience in years) of Each Survey Participant
Albania	1	Academia (0–9 years)
Austria	1	Clinical /Healthcare (10–19 years)
Belgium	1	Commercial company /Industry (20–29 years)
Bosnia and Herzegovina	2	Academia (10–19 years) Government /Health Administration /Health Authority (10–19years)
Bulgaria	2	Academia (0–9 years) Academia (≥30 years)
Croatia	2	Academia (10–19 years) Clinical /Healthcare (0–9 years)
Cyprus	1	Health Insurance /Regulatory Agency (20–29 years)
Czech Republic	1	Academia (≥30 years)
Denmark	1	Academia (20–29 years)
Estonia	2	Health Insurance /Regulatory Agency (10–19 years) Academia (20–29 years)
Finland	1	Academia (0–9 years)
France	1	Commercial company /Industry (0–9 years)
Germany	1	Academia (10–19 years)
Greece	1	Academia (0–9 years)
Hungary	2	Clinical /Healthcare (10–19 years) Other: Research /Education not Academia (0–9 years)
Iceland	2	Clinical /Healthcare (10–19 years) Clinical /Healthcare (10–19 years)
Ireland	2	Commercial company /Industry (0–9 years) Academia (≥30 years)
Israel	1	Academia (0–9 years)
Italy	1	Academia (10–19 years)
Latvia	1	Clinical /Healthcare (20–29 years)
Lithuania	2	Academia (20–29 years) Academia (10–19 years)
Luxembourg	1	Academia (0–9 years)
Malta	1	Academia (≥30 years)
Moldova	1	Academia (10–19 years)
Montenegro	2	Academia (20–29 years) Clinical /Healthcare (0–9 years)
Netherlands	1	Academia (10–19 years)
North Macedonia	1	Academia (10–19 years)
Norway	1	Academia (20–29 years)
Poland	1	Academia (20–29 years)
Portugal	3	Academia (≥30 years) Academia (20–29 years) Academia (0–9 years)
Romania	1	Academia (20–29 years)
Serbia	1	Academia (10–19 years)
Slovakia	1	Academia (20–29 years)
Slovenia	1	Clinical /Healthcare (20–29 years)
Spain	3	Other: Research /Education not Academia (≥30 years) Academia (10–19 years) Academia (0–9 years)
Sweden	1	Academia (20–29 years)
Switzerland	2	Academia (≥30 years) Academia (20–29 years)
Turkey	1	Academia (10–19 years)
United Kingdom	2	Other: Clinical Academia (≥30 years) Clinical /Healthcare (20–29 years)

### Reimbursed Medication Adherence Enhancing Interventions

**FIGURE 1:**
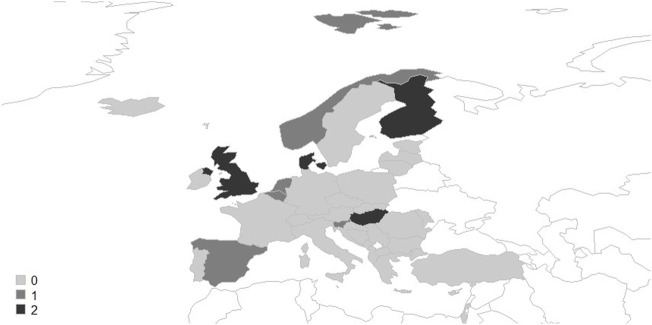
Number of reimbursed medication adherence enhancing interventions across European countries.

The survey identified 13 reimbursed MAEIs from nine countries (Figure 1). Interventions were categorized by the following types: multi-dose drug dispensing (MDD) (*n* = 5), medication review (*n* = 4), smart device (*n* = 2), mobile application (*n* = 1), and patient education (*n* = 1). We did not identify any MAEI planned to be reimbursed in the next 24 months in the evaluated countries. Characteristics of the analyzed MAEIs are summarized in Table 2.

**TABLE 2:** Characteristics of identified reimbursed medication adherence enhancing interventions.

Type of Intervention	Country	Year of Introduction	Level of Intervention	Target Population	Who Pays the Reimbursement?	Who Gets the Reimbursement?
Multi-dose drug dispensing	Belgium	2012	National	Elderly patients	Public insurance /Public healthcare system /Government	Pharmacy
	Denmark	2001	National	Elderly patients		
	Finland	2006	National	Reimbursed only for patients ≥75 years of age and using ≥6 drugs suitable for drug dispensing		
	Norway	Early 2010s	National	Elderly patients		
	United Kingdom	2014	National	Elderly patients, or those otherwise struggling to cope with their medication		
Medication review	Hungary	2019	National	40–65 years old patients with chronic disorders	Public insurance /Public healthcare system /Government	Primary care (GP)
	Slovenia	2016	National	Patients with drug related problems; identified and referred by a GP		Primary care (clinical pharmacist)
	Spain	2012	Regional	Patients with chronic diseases and polypharmacy		Primary care, Hospital and Pharmacy
	United Kingdom	Years ago	National	Patients on long-term medication		Pharmacy and Hospital
Smart device	Finland	2019	National	Patients on rheumatoid arthritis medication	Pharma company	IT company
	Netherlands	2020	National	Patients with asthma/COPD	Public insurance /Public healthcare system /Government and Pharma company	Pharmacy
Mobile application	Denmark	No information	National	Patients with mental disorder	No information	No information
Patient education	Hungary	2016	National	Newly transplanted patients	Patient organization	Healthcare providers

COPD, chronic obstructive pulmonary disease; GP, general practitioner; IT, information technology.

MDD services were implemented and reimbursed primarily in Northern and Western European countries (i.e., Belgium, Denmark, Finland, Norway, and the United Kingdom). In all countries MDD services were reimbursed by public health insurance predominantly to older people who take multiple medicines either at home or in nursing homes.

Based on our results, medication review was reimbursed in 4 European countries (i.e., Hungary, Slovenia, Spain, and the United Kingdom). In all but one of these countries this service was provided by primary care centers; in the United Kingdom community pharmacies were responsible for medication review. The identified medication review services were reimbursed by public health insurance primarily for patients with chronic disorders. In Slovenia, two types of medication reviews were available. The “type 3” medication review (PCNE, 2016) performed in primary care centers was reimbursed since 2016, while the “type 2a” medication review (PCNE, 2016) provided by community pharmacies was not reimbursed. In Hungary, from 2018 as part of the “Three Generations for Health Program” consortiums of primary care centers could get reimbursement for providing medication review type services; however, the program was closed at the end of 2021.

Experts from Finland and the Netherlands reported that in their countries there were reimbursed adherence enhancing smart devices. Popit Sense® is a smart device for monitoring pill-taking. The device monitors through sensors when pills are taken. Data on pill consumption are sent to Popit Pill Reminder Application® on a smartphone. This smart device was reimbursed by a pharma company in Finland for patients with rheumatoid arthritis. Another example is the Enerzair® smart inhaler which is a drug-device combination (devices integrated with a drug and dispensed at the same time). The device is connected with a mobile application for self-monitoring. This smart inhaler was reimbursed by the national health insurance in the Netherlands for the maintenance treatment of asthma/COPD in adult patients.

In our survey we identified only one reimbursed mobile health application for enhancing medication adherence. MindFrame® is a mobile health solution that supports the treatment of individuals suffering from schizophrenia in Denmark. This application helps patients to play a more active role in their treatment and allows mental health professionals to monitor patients remotely.

Last but not least, we identified one reimbursed patient education program as well. The “Be Educated and Empowered Patient” (BEEP) is an education program for organ transplanted patients launched by the Hungarian Transplant Federation. The program was reimbursed from various funds of pharma companies and state grants. This program primarily aims to improve the health literacy level and health behaviour of newly transplanted patients and thus it only has an indirect effect on medication adherence.

### Association Between Country Economy and the Availability of Reimbursed Medication Adherence Enhancing Interventions

**FIGURE 2:**
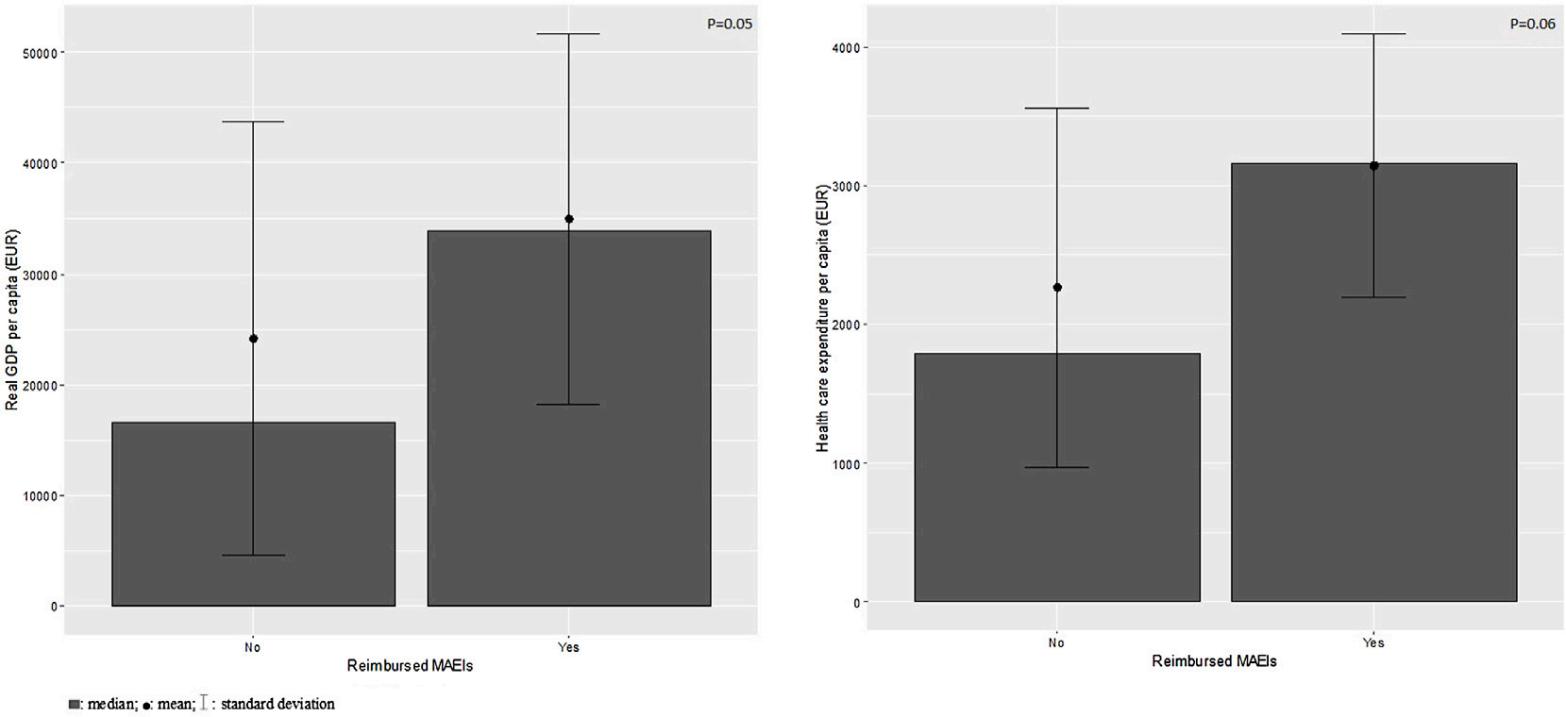
Association between country income and health care expenditure, and the availability of reimbursed medication adherence enhancing interventions across European countries. GDP: gross domestic product; MAEI: medication adherence enhancing intervention.

We found a significant difference between the median real GDP per capita (*p* = 0.05) for countries having ≥1 (33,880 EUR) compared to no reimbursed (16,620 EUR) MAEI (Figure 2). In case of median health care expenditure per capita the difference was statistically not significant (countries with ≥1 vs no reimbursed MAEI: 3,154 EUR vs 1,788 EUR, respectively; *p* = 0.06).

## Discussion

To our knowledge, this is the first study to provide an in-depth overview on reimbursed MAEIs across Europe. From the evaluated 39 countries, there were only nine countries in which we could identify reimbursed adherence interventions. Our findings confirm that despite of the considerable economic and clinical burdens of medication non-adherence, MAEIs remain on a low priority on the health policy agenda of funding bodies. In the European Union, almost 200,000 people die each year because of non-adherence and the direct and indirect costs of poor adherence were estimated to be 80–125 billion EUR annually (European Commission, 2011). However, these losses could be reduced by implementing MAEIs in the everyday clinical practice.

At present, there is no uniform terminology for MAEIs which made it difficult to identify reimbursed adherence interventions. In our survey, MAEI was defined by the authors as a result of discussion and consensus as “any structured intervention aiming to help patients to make optimal use of their pharmacotherapy”. However, it might be that respondents interpreted this definition differently when determining whether an intervention affects medication adherence or not. One uniform, common accepted, standard definition for MAEI would be highly warranted to be able to define interventions improving adherence more precisely; particularly in the view of the wide range of various types of educational (e.g., group/individual education provided by physicians, pharmacists, nurses, allied health professionals) and behavioural (e.g., calendar/diary, reminder chart/medication list, large print labels, packaging change, multi-compartment pillbox/calendar pack/compliance aid, adherence monitoring, reminders) interventions developed recently (Cross et al., 2020).

In total, 13 reimbursed MAEIs were included in our analysis from which MDD and medication review were the most common. In general, as part of MDD, medicines such as tablets, capsules and pills are repackaged with a special equipment automatically into unit-dose bags according to the time of administration, then these bags are dispensed by the community pharmacy to the patient. Unit-dose bags are labelled with the patient’s identification data, the drug name, and time of administration (Sinnemaki et al., 2013; Rechel, 2018). Although several Northern and Western European countries embraced MDD to improve medication adherence, evidence on its cost implications is still limited (Rechel, 2018). Herborg et al. (Herborg et al., 2008) conducted a HTA for MDD in Denmark, but this analysis did not cover all HTA aspects. Their study was limited to stakeholders’ perspectives and perceptions on the implementation, operation, consequences, and future potential of MDD in the primary care; however, cost-effectiveness of MDD was not evaluated. This HTA concluded that MDD can be effective to improve the medication adherence of chronic patients in the Danish primary care, but there might be organizational obstacles (e.g., resistance from nurses and doctors). Medication review as defined by the Pharmaceutical Care Network Europe (PCNE) is “a structured evaluation of a patient’s medicines with the aim of optimizing medicines use and improving health outcomes” (PCNE, 2016). The medication review consultations between doctors, nurses or pharmacists and patients in primary care centers or community pharmacies help to increase patients’ knowledge and understanding of their pharmacotherapy and provide an opportunity to detect any drug-related problems. Regarding medication review, a recent meta-analysis found that even on a short-term period, this service has an effect on most drug-related outcomes (e.g., the number of drug changes, the number of drug-related problems, medication adherence); however, similar to MDD the available information does not allow to draw clear conclusions about its economic impact (Huiskes et al., 2017).

Other types of MAEIs such as e-health technologies (e.g., smart devices, mobile applications) or patient education programs were reimbursed only in limited number of European countries. Nevertheless, several e-health interventions have been developed in the past few years (Ma et al., 2022) which could provide an opportunity to improve medication adherence with minimal effort from health care providers whose time and resources are limited (Pouls et al., 2021).

Based on these findings we can conclude that although several studies have demonstrated that MAEIs may improve clinical outcomes (Salema et al., 2011; Nieuwlaat et al., 2014; van Driel et al., 2016; Blakey et al., 2018; Godinho et al., 2020; Zijp et al., 2020; Gohil et al., 2021; Whiteley et al., 2021), existing evidence on the economic aspects of MAEIs is of poor quality (Elliott et al., 2005). Heterogeneity in the results of economic evaluations within different intervention types is significant due to disparity in the nature of interventions, investigated outcomes, the measures of non-adherence used and time horizons of studies, which makes comparing findings challenging. It should be also noted, that different type of MAEIs may require different type of economic evaluations. For example, in case of service-based interventions (e.g., pharmacy services) multiple phases of the implementation process have to be taken into consideration (i.e., installation phase: preparation of the service provider to deliver the service, initial implementation phase: to pilot the service in a small number of patients, and full operation phase: the full implementation of the service in routine care) (Perraudin et al., 2019), in comparison to e-health technologies (e.g., smart devices) which can be evaluated in a conventional cost-effectiveness analysis. Besides clinical and economic impacts, the consideration of other factors, including social (e.g., access for vulnerable population groups, caregiver burden), and patient related factors (e.g., responsiveness to patients’ individual needs) during the critical evaluation of MAEIs may also facilitate decision making while allocating scarce resources. Additionally, the thorough HTA of an e-health intervention may require further specific aspects, e.g., software update and data privacy (Moshi et al., 2018). Lack of published evidence on the HTA and reimbursement pathways of MAEIs from other regions (e.g., North America, Asia) did not allow the comparison between regions. Using structured and explicit approaches for health policy decisions involving multiple value criteria during the HTA of MAEIs could help to identify the most effective interventions based on the best available evidence. Detailed recommendations on the value criteria and economic evaluations would help removing barriers relating to the HTA of MAEIs.

The majority (77%) of the identified MAEIs were reimbursed from public health care funds; however, improving medication adherence is a common goal of all stakeholders in the health care system (i.e., policy makers, pharma industry, health care providers, pharmacists, patients and caregivers). A close cooperation of key stakeholders related to the reimbursement of MAEIs could add a surplus value to the implementation by bridging the gap between clinical research and clinical practice.

We found a statistically significant association between country income (i.e., real GDP per capita, *p* = 0.05) and the availability of reimbursed MAEIs, and a not significant trend in case of health care expenditure. This result raises the possibility that not only the awareness of decision makers on medication non-adherence, but country income might also influence the implementation and reimbursement of MAEIs. Evidence suggests that MAEIs are usually not embedded in a broader understanding of the reasons for suboptimal adherence (Clyne and McLachlan, 2015). Further studies are needed to raise stakeholders’ awareness on medication non-adherence to overcome this challenge.

Our results should be considered in the light of certain limitations. First, participants’ answers to the survey may be biased by their subjectivity, background and work experience. The survey was completed by ENABLE members and in some countries, information was based on the answers of only one participant. The majority of respondents had academic background (i.e., medical or pharmaceutical sciences) and they might not have sufficient information on e.g. specific MAEIs reimbursed by pharma companies to patients with certain diseases only. Furthermore, it should be noted that the lack of a common definition for MAEIs might also bias the identification of reimbursed interventions. Although our survey might not provide a complete picture on the reimbursement landscape of MAEIs in Europe, it does provide a useful starting point for discussion and may also help to determine where further research is needed. Finally, our survey questionnaire with many closed questions allowed us to capture very specific information on MAEIs. To minimize the potential risks of the self-developed questionnaire, external experts were asked to assess its validity and technical functionality.

In conclusion, to date only a small number of MAEIs have been reimbursed across Europe. Discussions about MAEIs is hampered by the lack of a common terminology. Besides the clinical studies, more research effort should be devoted to better understand the effect of MAEIs on economic outcomes. Specific HTA process guidelines involving multiple value indicators and consequently the comprehensive assessment of MAEIs would help to identify the most effective and cost-effective adherence programs. A close cooperation of key stakeholders related to the reimbursement of MAEIs could set new benchmark to manage medication non-adherence.

## European Network to Advance Best Practices and Technology on Medication Adherence (Enable)

Sarah Wamala Andersson, Manon Belhassen, Katharina Blankart, Martina Bago, Job F. M. van Boven, Dragana Drakul, Natasa Duborija-Kovacevic, Lallas Efthymios, Dalma Erdősi, Nuria Garcia-Agua Soler, Bettina Husebo, Ivett Jakab, Barbora Kostalova, Jesper Kjærgaard, Dusanka Krajnovic, Fatjona Kamberi, Ott Laius, Francisca Leiva-Fernandez, Urska Nabergoj Makovec, Paulo Moreira, Zornitsa Mitkova, Panayiotis Petrou, Mitar Popovic, Marie Paule Schneider Voirol, Vesna Vujic-Aleksic, Bruno Velescu, Daisy Volmer, and Martin Wawruch

## Data Availability

The raw data supporting the conclusion of this article will be made available by the authors, without undue reservation.
