# Supplementary material for: Reimbursed Medication Adherence Enhancing Interventions in European Countries: Results of the EUREcA Study
Source: Front Pharmacol. 2022 Jun 17;13:892240. doi: 10.3389/fphar.2022.892240 (PMC9247400; doi:10.3389/fphar.2022.892240)
Supplement: Supplementary file 1 [file DataSheet1.PDF]

EUREcA Survey

1. Introduction & informed consent

Dear ENABLE COST Action Member,

Hereby, we invite you to take part in an important initiative called **EUREcA** (**EU**ropen **RE**imbursement strategies for interventions targeting medi**c**ation **A**dherence) performed by ENABLE COST Action WG3.

As a part of EUREcA, this anonymous survey is going to assess the availability of **reimbursement frameworks for medication adherence enhancing interventions across European countries, namely what kind of interventions exist, for which patients and at what level (i.e. regional or national level).**

In this survey, by ***"medication adherence enhancing intervention"*** **we understand any structured intervention, aiming to help patients to make optimal use of his/her pharmacotherapy.**

Our primary objective is to give an overview on reimbursed medication adherence enhancing interventions currently available in European countries. Please also note that EUREcA is collecting data at **regional and national levels only**, i.e. the scenario that is typical for your entire country or region (interventions run by single institutions, single-centered initiatives, clinical trials or research projects should not be considered). Furthermore, it is also important to highlight that in interventions of interest, medication adherence enhancement has to be in the primary focus (e.g. medication adherence apps) or at least it should be an element of a complex healthcare program (e.g. pharmacy-led disease management program). Interventions can be reimbursed by public healthcare systems, governments, public or private insurances, pharma companies, patient organizations, or other organizations as well.

The average time required to answer the survey is estimated at 20 minutes.

Practical hints:

1. Please provide your answers **as per June, 2021**.
2. This questionnaire provides the space for detailed description of up to 3 interventions. Please choose the most important ones.
3. Be honest and do not overestimate the small local initiatives, even if you do not know any reimbursed medication adherence enhancing interventions in your country. We can believe that currently, in many European countries such instruments are lacking.

Should you have any questions related to the questionnaire, please email Tamas Agh at tamas.agh@syreon.eu.

Thank you in advance for your prompt and accurate answer!

EUREcA Steering Committee

* 1. We kindly ask you to provide informed consent with your participation in this study:

- I have read the information for the respondent, I understand the formal aspects that it involves, that my participation is voluntary and that I can withdraw or request that my data be withdrawn whenever I want, without having to give explanations and without affecting my professional activity. Contact person for withdrawing informed consent and data: Tamas Agh tamas.agh@syreon.eu .
- I understand that I will not receive financial compensation for my participation in the study.
- I understand that the information will be confidential and that no unauthorized person will have access to the data.
- I understand that analysis of compiled answers will be published.
- I know how to contact the investigators.

Thank you for your participation!

Informed consent (mandatory field):

○ I agree to participate voluntarily in the study and with the use of my anonymized data for purposes and outputs in the survey **EUREcA** (EUropen REimbursement strategies for interventions targeting medication Adherence)

○ No, I don't want to participate in the study

EUREcA Survey

2. Your data

* 2. Which country do you represent?

Country [ ]

* 3. What is your primary field of work?

- ◯ Clinical / Healthcare
- ◯ Academia (research or education)
- ◯ Health Insurance / Regulatory Agency
- ◯ Government / Health Administration / Health Authority
- ◯ Commercial company / industry
- ◯ Other (please specify below)

Other (please specify)

[ ]

* 4. What is your overall work experience in your primary field of work (in years)?

- ◯ 0-9
- ◯ 10-19
- ◯ 20-29
- ◯ 30+
- ◯ Prefer not to answer

EUREcA Survey

3. Availability of reimbursed medication adherence enhancing interventions

* 5. Are you aware of any **reimbursed** medication adherence enhancing intervention(s) introduced in your country?

- ◯ Yes
- ◯ No

EUropen REimbursement strategies for interventions targeting medication Adherence

EUREcA Survey

4. Reimbursed interventions

* 6. What is the name of this **reimbursed** medication adherence enhancing intervention? Please provide name in English and the native language as well.

* 7. **Who pays** for this intervention (choose all that apply)

- [ ] Public insurance, Public healthcare system, Government
- [ ] Private insurance
- [ ] Pharma company
- [ ] Patient organizations
- [ ] Patient (the intervention is only partly reimbursed, and patient co-payment is required)
- [ ] Others - please specify below
- [ ] I do not know

Other (please specify)

* 8. **Who gets the payment** for this intervention (choose all that apply)

- [ ] Pharmacy (pharmacists)
- [ ] Primary Health Care center (family physicians, nurses)
- [ ] Out-patient clinic (physicians, nurses)
- [ ] Hospital (physicians, nurses)
- [ ] Pharma company
- [ ] IT company
- [ ] Patient organizations
- [ ] Others - please specify below
- [ ] I do not know

Other (please specify)

* 9. Please provide a short description of the type of this reimbursed medication adherence enhancing intervention.

Type of intervention: Patient education and counselling programs directly aimed to enhance medication adherence (e.g. health promotion programs for chronic patients) - please specify:

Type of intervention: Structured evaluation of a patient's medicines in order to optimize medicines use (e.g. medication use review, medication therapy management, medication regimen simplification) - please specify:

Type of intervention: Interventions supported by new digital health technology available on your native language (i.e. medication adherence apps) - please specify:

Other type of intervention: - please specify:

* 10. Please provide additional information on this reimbursed medication adherence enhancing intervention.

Target patient group (e.g. disorder, age group): [ ]

Other relevant information (e.g. contact data for the intervention): [ ]

If available, please provide a link to external description (English or native language): [ ]

* 11. When was this reimbursed medication adherence enhancing intervention introduced? Please provide the year.

[ ]

* 12. What is the level of this intervention?

- ○ National
- ○ Regional

* 13. Is the effectiveness of this reimbursed medication adherence enhancing intervention monitored? (i.e. are there any goal setting and outcome monitoring employed) (choose all that apply)

- ☐ Yes; outcome monitored = Medication adherence – please specify below
- ☐ Yes; outcome monitored = Specific clinical / health outcome(s) – please specify below
- ☐ Yes; outcome monitored = Resource utilization (e.g., emergency department utilization, hospitalization) – please specify below
- ☐ Yes; outcome monitored = Healthcare costs – please specify below
- ☐ Yes; outcome monitored = Other – please specify below
- ☐ No
- ☐ I do not know

Other (please specify)

[ ]

* 14. What is the basis for reimbursement of this reimbursed medication adherence enhancing intervention? (choose all that apply)

- ☐ Per capita payment
- ☐ Fee for service
- ☐ Other – please specify below
- ☐ I do not know

Other (please specify)

[ ]

* 15. Are you aware of any other **reimbursed** medication adherence enhancing intervention(s) introduced in your country?

- ◯ Yes
- ◯ No

ENABLE ADHERENCE

EUREcA ENABLE ADHERENCE — EUropen REimbursement strategies for interventions targeting medication Adherence

EUREcA Survey

5. Reimbursed interventions (2)

* 16. What is the name of this **reimbursed** medication adherence enhancing intervention? Please provide name in English and the native language as well.

* 17. **Who pays** for this intervention (choose all that apply)

- ☐ Public insurance, Public healthcare system, Government
- ☐ Private insurance
- ☐ Pharma company
- ☐ Patient organizations
- ☐ Patient (the intervention is only partly reimbursed, and patient co-payment is required)
- ☐ Others - please specify below
- ☐ I do not know

Other (please specify)

* 18. **Who gets the payment** for this intervention (choose all that apply)

- [ ] Pharmacy (pharmacists)
- [ ] Primary Health Care center (family physicians, nurses)
- [ ] Out-patient clinic (physicians, nurses)
- [ ] Hospital (physicians, nurses)
- [ ] Pharma company
- [ ] IT company
- [ ] Patient organizations
- [ ] Others - please specify below
- [ ] I do not know

Other (please specify)

____

* 19. Please provide a short description of the type of this reimbursed medication adherence enhancing intervention.

Type of intervention: Patient education and counselling programs directly aimed to enhance medication adherence (e.g. health promotion programs for chronic patients) - please specify: ________________

Type of intervention: Structured evaluation of a patient's medicines in order to optimize medicines use (e.g. medication use review, medication therapy management, medication regimen simplification) - please specify: ________________

Type of intervention: Interventions supported by new digital health technology available on your native language (i.e. medication adherence apps) - please specify: ________________

Other type of intervention: - please specify: ________________

* 20. Please provide additional information on this reimbursed medication adherence enhancing intervention.

Target patient group (e.g. disorder, age group):

Other relevant information (e.g. contact data for the intervention):

If available, please provide a link to external description (English or native language):

* 21. When was this reimbursed medication adherence enhancing intervention introduced? Please provide the year.

* 22. What is the level of this intervention?

- National
- Regional

* 23. Is the effectiveness of this reimbursed medication adherence enhancing intervention monitored? (i.e. are there any goal setting and outcome monitoring employed) (choose all that apply)

- Yes; outcome monitored = Medication adherence – please specify below
- Yes; outcome monitored = Specific clinical / health outcome(s) – please specify below
- Yes; outcome monitored = Resource utilization (e.g., emergency department utilization, hospitalization) – please specify below
- Yes; outcome monitored = Healthcare costs – please specify below
- Yes; outcome monitored = Other – please specify below
- No
- I do not know

Other (please specify)

* 24. What is the basis for reimbursement of this reimbursed medication adherence enhancing intervention? (choose all that apply)

- Per capita payment
- Fee for service
- Other – please specify below
- I do not know

Other (please specify)

* 25. Are you aware of any other **reimbursed** medication adherence enhancing intervention(s) introduced in your country?

- ◯ Yes
- ◯ No

ENABLE ADHERENCE

EUREcA ENABLE ADHERENCE — EUropen REimbursement strategies for interventions targeting medication Adherence

EUREcA Survey

6. Reimbursed interventions (3)

* 26. What is the name of this **reimbursed** medication adherence enhancing intervention? Please provide name in English and the native language as well.

[ ]

* 27. **Who pays** for this intervention (choose all that apply)

- ☐ Public insurance, Public healthcare system, Government
- ☐ Private insurance
- ☐ Pharma company
- ☐ Patient organizations
- ☐ Patient (the intervention is only partly reimbursed, and patient co-payment is required)
- ☐ Others - please specify below
- ☐ I do not know

Other (please specify)

[ ]

* 28. **Who gets the payment** for this intervention (choose all that apply)

- [ ] Pharmacy (pharmacists)
- [ ] Primary Health Care center (family physicians, nurses)
- [ ] Out-patient clinic (physicians, nurses)
- [ ] Hospital (physicians, nurses)
- [ ] Pharma company
- [ ] IT company
- [ ] Patient organizations
- [ ] Others - please specify below
- [ ] I do not know

Other (please specify)

____

* 29. Please provide a short description of the type of this reimbursed medication adherence enhancing intervention.

Type of intervention: Patient education and counselling programs directly aimed to enhance medication adherence (e.g. health promotion programs for chronic patients) - please specify: __________

Type of intervention: Structured evaluation of a patient's medicines in order to optimize medicines use (e.g. medication use review, medication therapy management, medication regimen simplification) - please specify: __________

Type of intervention: Interventions supported by new digital health technology available on your native language (i.e. medication adherence apps) - please specify: __________

Other type of intervention: - please specify: __________

* 30. Please provide additional information on this reimbursed medication adherence enhancing intervention.

Target patient group (e.g. disorder, age group): [ ]

Other relevant information (e.g. contact data for the intervention): [ ]

If available, please provide a link to external description (English or native language): [ ]

* 31. When was this reimbursed medication adherence enhancing intervention introduced? Please provide the year.

[ ]

* 32. What is the level of this intervention?

- National
- Regional

* 33. Is the effectiveness of this reimbursed medication adherence enhancing intervention monitored? (i.e. are there any goal setting and outcome monitoring employed) (choose all that apply)

- [ ] Yes; outcome monitored = Medication adherence – please specify below
- [ ] Yes; outcome monitored = Specific clinical / health outcome(s) – please specify below
- [ ] Yes; outcome monitored = Resource utilization (e.g., emergency department utilization, hospitalization) – please specify below
- [ ] Yes; outcome monitored = Healthcare costs – please specify below
- [ ] Yes; outcome monitored = Other – please specify below
- [ ] No
- [ ] I do not know

Other (please specify)

[ ]

* 34. What is the basis for reimbursement of this reimbursed medication adherence enhancing intervention? (choose all that apply)

- [ ] Per capita payment
- [ ] Fee for service
- [ ] Other – please specify below
- [ ] I do not know

Other (please specify)

[ ]

* 35. Are you aware of any other **reimbursed** medication adherence enhancing intervention(s) introduced in your country?

- ◯ Yes (if so, please specify below)
- ◯ No

Please specify here these interventions

[ ]

EUREcA Survey

Public discussion on medication interventions

36. Are there any plans to introduce in your country or region any reimbursed medication adherence enhancing interventions in **the next 24 months**? If so, please provide a short description of these plans.

[ ]

EUREcA Survey

Final questions & Thank you!

37. Would you like to provide us with any additional comments?

[ ]

Thank you very much for completing the EUREcA survey!

With your answer, we hope to help sustainability of European healthcare systems.
